# Is the relationship among outcome variables shown in randomized trials?

**DOI:** 10.1186/s13063-015-0584-6

**Published:** 2015-02-22

**Authors:** David L Schriger, Richelle J Cooper, Ana Lopez-O’Sullivan, Carter Wystrach, Douglas G Altman

**Affiliations:** Department of Emergency Medicine, University of California, 924 Westwood Blvd, Suite 300, Los Angeles, CA 90024 USA; Centre for Statistics in Medicine, Nuffield Department of Orthopaedics, Rheumatology and Musculoskeletal Sciences, University of Oxford, Windmill Road, Oxford, OX3 7LD UK

**Keywords:** Journalology, Clinical trials, Reporting guidelines, Clinical outcomes

## Abstract

**Background:**

Randomized controlled trials (RCTs) often have more than one primary outcome and frequently have secondary and harm outcomes. Comparison of outcomes between study arms is the primary focus of RCTs, but there are times when the relation between outcomes is important, such as determining whether an intermediate outcome and a clinical outcome have a strong association. We sought to determine how often reports of RCTs depict the relations among outcomes at the individual patient level and, for those studies that use composite outcomes, how often the relations between component elements are depicted.

**Methods:**

We selected 20 general, specialty and subspecialty medical journals with high impact factors that publish original clinical research. We identified every RCT in the 2011 and 2012 issues and randomly selected 10 articles per journal. For each article we recorded the number of outcomes, the number of composite outcomes and how often the relations between outcomes or elements of composite outcomes were portrayed.

**Results:**

All but 16 of the 200 RCTs had more than one outcome. Thus, outcomes could have been related in 92% of studies, but such relations were only reported in 2 (1%). A total of 33 (17%) investigations measured a composite outcome, 32 of which showed data for each component. None, however, showed cross-tabulation of the components.

**Conclusions:**

Readers are rarely shown the relation between outcomes. Mandatory posting of datasets or requirements for detailed appendices would allow readers to see these cross-tabulations, helping future investigators know which outcomes are redundant, which provide unique information and which are most responsive to changes in the independent variables. While not every relationship between outcomes requires depiction, at present such information is seldom portrayed.

**Electronic supplementary material:**

The online version of this article (doi:10.1186/s13063-015-0584-6) contains supplementary material, which is available to authorized users.

## Background

Ethical considerations mandate that maximal use is made from the data obtained in randomized trials of humans and animals [1-3]. While many have called for the public availability of all such data, concerns about patient confidentiality and investigator reluctance to cede control of the data they worked hard to collect, have made progress in this direction slow. There are, however, many ways to share more data within a traditional publication format that are not currently being fully exploited. Only a fraction of available data about trial outcomes is conveyed in the typical trial report [4], a problem that can be lessened by including better tables and figures [5,6].

We have noticed that studies that report on several outcomes seldom provide information on the relation between these outcomes. Similarly, studies that use composite outcomes seldom compare the elements of the composite at the level of the individual. There are circumstances when this could be desirable. For example, future researchers may wish to know how well an easily or inexpensively obtained secondary outcome relates to a more clinically important, patient-centered outcome that is more difficult or costly to obtain. Such knowledge could help in the selection of outcomes in future trials. Knowledge of such relationships at the individual level can also be useful in providing construct validity to a trial and helping investigators understand the mechanisms by which an intervention works. The conclusions of a pediatric asthma study that showed that one inhaler reduced days missed from school (the primary clinical outcome) more than another would be different if there was a strong negative correlation between days missed from school and improvement in forced expiratory volume (FEV-1) than if there was a strong positive correlation. The latter would suggest that improvement in airflow was not on the causal pathway from inhaler use to better school attendance, requiring investigators to check their data for bias or to revise their theoretical model.

We conducted this study to determine how often investigators reported the relationship between outcomes in their studies for both individual outcomes and for the components of composite outcomes.

## Methods

### Study design

We performed a cross-sectional survey of randomized controlled trials (RCTs) published in 20 general medical, specialty and subspecialty journals with high impact factors between 2011 and 2012.

### Selection of journals

We chose the top journals based on the 2011 Journal Citation Reports Impact Factor [7], selecting journals that publish original clinical research in English and excluding those that publish only review articles or predominantly basic science research. We selected the six highest ranked general medical journals, the highest ranked journal in seven specialties and the highest ranked journal in seven randomly selected medical and surgical subspecialties (list of journals in Additional file 1: Table S1).

#### Selection of articles within journals

We identified every RCT in the 2011 and 2012 issues of each journal by searching MEDLINE for publication type ‘Randomized Controlled Trial’ or a title with ‘random*’. For each journal we generated a randomly ordered list of RCTs and selected the first 10 that were primary reports of studies of at least 40 humans. We used a separate sample of 20 RCTs from 2012 issues of general medical journals to develop and refine the data collection and coding rules.

### Data abstraction

We reviewed the entire RCT report including all print and online supplements and appendices using a data collection instrument we developed for this study. After developing a preliminary scoring form and coding manual, four authors each independently coded 10 RCTs and met to refine variable definitions and coding rules. Two authors then scored another 10 RCTs, inter-rater reliability was assessed and rules were further revised. These authors then each scored half of the 200 RCTs included in the final data set. Both raters independently scored 10 papers as a final formal measure of inter-rater reliability.

### Variable definition

The data form contained entries for: the number of primary, secondary and harm outcomes reported in the study; the number of composite outcomes and whether components were separately reported; and the number of outcomes and components that were compared to each other at the subject level.

For each RCT we determined if the authors either explicitly (‘the primary outcome was…’) or implicitly (for example ‘we based our sample size on …’) identified a primary outcome, and if so, how many outcomes were identified as ‘primary’. We also counted the number of secondary outcomes and the number of harm outcomes, considering an outcome as a ‘harm’ whenever the authors or context deemed it so. Any outcome not categorized as primary or harm was considered secondary, unless there were no explicitly or implicitly stated primary outcomes, in which case all non-harm outcomes were considered primary.

To avoid over-counting, if several secondary outcome variables each measuring aspects of the same construct (for example FEV-1, peak flow and minute ventilation, all measuring airflow) were measured at multiple times (for example at four different times), we did not count this as 12 secondary outcomes but instead counted either the number of variables (three in this case) or the number of measurements of each variable (four), whichever was the larger number (four in this example). When investigators grouped harms either by severity (for example mild versus moderate versus severe) or organ system (for example cardiac or pulmonary) or another rubric, we counted the number of categories rather than the number of individual harm variables. Having identified and enumerated all primary, secondary and harm outcomes, we then identified any comparison or cross-tabulation of the outcomes at the subject level, and categorized each as primary versus primary, primary versus secondary, primary versus harm and so forth.

#### Primary analysis

Our analysis was descriptive. The unit of analysis was the research paper, and our primary outcome was the number of articles reporting a relationship among outcomes, by which we mean any graph, table or text that provided an indication of how one outcome variable related to another at the individual patient level. Data were collected in a customized spreadsheet that included data checking rules, and analyses were performed with Stata 12 software (Stata Corp, College Station, Texas, United States). We recorded each journal’s impact factor and whether the journal endorsed the Consolidated Standards of Reporting Trials (CONSORT) reporting guidelines by reviewing their Instructions for Authors and journal website information [8].

#### Post hoc analysis

*Trials* peer reviewers suggested that the comparisons we sought might be presented in secondary trial publications rather than the main publication of the trial. To examine this hypothesis we randomly selected one article per journal from our 200 article database and checked MEDLINE for papers citing the article. We then checked these citations to see if any were secondary reports of the original RCT and, if so, if they contrasted any of the outcomes from the primary study.

## Results

The 2011 impact factor of the 20 journals in our study ranged from 3.3 to 53.3. All but one endorsed the CONSORT statement. (Additional file 1: Table S1) We reviewed 224 articles to identify the 200 articles (10 per journal) in our sample. We excluded 24 articles (16 that were not primary reports of RCTs, four research letters, one pilot study (<40 subjects), one nonhuman subjects, one cost analysis of an RCT and one editorial). The final round of inter-rater reliability of abstraction achieved 100% agreement on all items except the number of harms (80%, eight out of 10). After clarifying the scoring rule, the harm items in the 10 articles were rescored and agreement was 100%; raters then rescored the harm items in all articles they had scored in order to ensure consistency.

**Figure 1:**
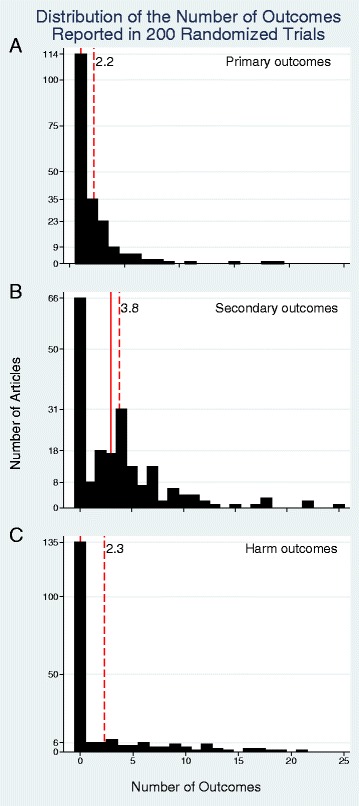
**Histogram of the number of outcomes reported in the 200 randomized controlled trials. (A)** Primary outcomes; **(B)** Secondary outcomes; **(C)** Harm outcomes. Solid red line = median. Dashed red line = mean.

All but two studies identified a primary outcome; in one the authors stated that they intentionally avoided considering any of the 11 outcomes primary, in the other there were six outcomes and no comment regarding which was primary [9,10]. While the word primary implies that there would be a single most important outcome, the CONSORT statement recommends a single primary outcome and study size calculations are almost always based on a single outcome, 43% (86 out of 200) of articles had more than one primary outcome [11]. The mean number of primary outcomes was 2.2 (median: 1, range: 1 to 19) (Table 1, Figure 1A). A total of 134 (67%) studies reported secondary outcomes and 64 (32%) reported harms. Across all studies, a mean of 3.8 secondary outcomes and 2.4 harms were reported. (Figure 1B, C). Studies that reported at least one secondary outcome reported a mean of 5.7 (median: 4, range: 1 to 25). For harms these numbers were 7.3 (median: 6, range: 1 to 21).

**Table 1:** **Outcome reporting in 200 randomized trials**

**Trial outcomes (n = 200)**	
Primary outcome stated explicitly	198
Number of primary outcomes: mean, median, (range) (IQR)	2.2, 1, (1, 19) (1, 3)
Papers with one primary outcome: N (%)	114 (57%)
Number of secondary outcomes: mean, median, (range) (IQR)	3.8, 3, (0, 25) (0, 5)
Number of harms: mean, median, (range) (IQR)	2.4, 0, (0, 21) (0, 3)
Papers with more than one outcome: N (%)	184 (92%)
Comparisons among outcomes (n = 184)	
Number of papers with comparisons between outcomes	2
Total number of comparisons between outcomes	28
Type of comparison	
Primary versus primary	1
Primary versus secondary	11
Primary versus harm	6
Secondary versus secondary	4
Secondary versus harm	6
Harm versus harm	0
Composite primary outcomes: N (%)	33 (17%)
Number of items in composites: mean, median (range) (IQR)	4.7, 3, (2, 21) (2, 5)
Number of papers showing individual elements: N (%)	32/33 (97%)
Number of individual elements shown: N (%)	152/155 (98%)
Individual elements compared to other individual elements	0

Among the 184 (92%) studies that reported more than one outcome, only two (1%) presented the relationship between outcomes. These two articles reported 28 different relationships (Table 1). One article in the *American Journal of Psychiatry* was a placebo controlled trial of metformin to reverse amenorrhea in schizophrenics [12]. The primary outcome measures were: the percentage of women who had restoration of menstruation, change in body weight and change in Body Mass Index (BMI). The secondary measures were several hormone levels and a symptom score, the Positive and Negative Syndrome Scale (PANSS). In addition to reporting outcome measures for each group, the authors performed a logistic regression to examine whether changes in body weight and BMI (both primary outcomes) and five hormone levels (secondary outcomes) were predictive of return of menstruation (primary outcome). By so doing they attempted to elucidate the mechanism by which metformin might help restore menstruation.

The second paper, in *The Lancet*, reported an RCT comparing defibrotide and no treatment for the prevention of hepatic veno-occlusive disease in children undergoing stem cell transplant [13]. The primary outcome was the occurrence of a hepatic veno-occlusive event in the 30 days post-transplant. Secondary outcomes included 100 and 180-day mortality, multiorgan failure and graph versus host disease. Harm outcomes included sepsis and cardiovascular and pulmonary complications. The relationships among these variables were presented in a supplementary table which contained 26 comparisons (10 primary versus secondary, four secondary versus secondary, six harm versus primary and six harm versus secondary).

A total of 17% of the RCTs (33 out of 200) reported a composite outcome, with a mean of 4.7 elements (median: 3, range: 2 to 21) per composite. All but one article (97%, 32 out of 33) reported results for the individual elements of the composite (152 of 155 elements). None of the articles depicted the relationship among components.

In our *post hoc* analysis we found that 14 of the 20 original trial reports had no secondary publications. For the six trials with secondary publications, none contained contrasts among outcome variables. While it is likely that we would find a few comparisons in secondary studies of the 180 trials we did not check for secondary publications, their numbers will be insufficient to change our conclusions, and it did not seem worth the effort to check the other 180 papers in the name of completeness.

## Discussion

Our study demonstrates that authors almost never (1%) present the relationship between outcomes of randomized trials in primary reports in medical journals with high impact factors. We did not attempt to score how often this truly represented a ‘missed opportunity’ as this concept is highly subjective and is dependent on the reader and context. Nevertheless, there are certainly some papers for which some readers would have been interested in the relationship among outcome variables at the patient level. Such relations could be reported in an online-only appendix so they are available to the interested reader but are not distracting to others. As noted in our Background section, an understanding of such relationships can assist in the selection of outcome measures for future research, provide construct validity (for example, when biomarkers relate to clinical outcomes in expected ways) and provide insight into the mechanism of action of an intervention (as in the aforementioned *American Journal of Psychiatry* paper). We note, however, that care is needed in conducting and interpreting relationships between outcomes within a trial. Our focus has been on associations. For analyses relating intermediate outcomes to final outcomes, however, there may be a temptation to seek a causal explanation. Such inference requires complex considerations, beyond the scope of this paper [14]. For example, misinterpretation is likely when relating a clinical outcome to compliance with therapy or to tumor response in cancer trials [15,16].

It is difficult to identify articles that contrast outcomes using MEDLINE as there are no MeSH (Medical Subject Heading) or study design terms that can be used to flag such papers. We had better results searching Google Scholar using ‘randomized trial’ and ‘correlation of change’, a strategy that identified a number of articles that contrasted outcomes. Many of these articles were secondary analyses of larger trials; studies that would not have been included in our primary study [17-19]. Others were exactly what we envisioned. Lapperre *et al*. compared treatments for chronic obstructive pulmonary disease (COPD) in smokers measuring both inflammatory cell counts and intermediate clinical outcomes [20]. Their figure four contrasts these outcomes, and shows that changes in cluster of differentiation 4 (CD4) and mast cell counts are poorly correlated with changes in FEV-1 and methacholine challenge [20]. Krasner *et al*. provided primary care physicians with a mindfulness education program, conducting assessments of mindfulness, burnout, empathy and psychosocial orientation and mood, before and several times after the intervention [21]. They compared these different outcomes using correlation (their table four) and demonstrated that an increase in mindfulness was associated with a decrease in burnout scores and mood disturbance scales and an increase in the quality of empathy [21]. In a secondary analysis of the CLEVER (Claudication: Exercise Vs. Endoluminal Revascularization) trial, Murphy *et al*. compared revascularization and supervised exercise for the treatment of leg claudication and demonstrated that the degree of correlation between clinical outcomes is different in the two treatment groups, a very important finding for those doing research in this field [17].

For composite outcomes, an understanding of the relationship among the components at the by-subject level may be as important as knowing the frequency of each component. All but one journal in our study endorsed CONSORT, but CONSORT does not mention this aspect of trial reporting [8]. If the CONSORT group decide that authors should depict the relationships among key outcomes, then the addition of an item to the CONSORT document would likely increase this aspect of trial reporting. Many have called for more transparency in the reporting of clinical trials, including the electronic posting of data sets so that researchers can fully evaluate authors’ claims and make full use of the data [1-3]. Until such time as this practice becomes routine, depiction of the relationships between trial outcome measures in supplementary tables and figures may help to maximize the information gained from these trials.

We evaluated articles in journals with high impact factors; results in other journals may differ. There is little reason to believe that relationships between outcomes would be more frequently reported in such journals. We evaluated only 10 articles per journal, a number too small to ensure stable estimates for each journal. While our overall estimate of 1% may be somewhat imprecise, our conclusion, that such depictions are rare, is not at risk. Finally, we did not evaluate secondary reports of all the RCTs. It is conceivable that there were some relationships among outcomes presented there.

## Conclusions

Authors seldom present comparison of trial outcomes, a practice that is quite important in selected circumstances. This finding is yet another demonstration of the clinical research community’s failure to fully utilize the data generated from clinical trials: a breakdown in the moral contract between researchers and the patients who consent to participate in such activities.

## Additional file

Additional file 1: Table S1. Impact Factor and CONSORT status of study journals.

## Abbreviations

BMI: Body mass index
CD4: Cluster of differentiation 4
CONSORT: Consolidated standards of reporting trials
COPD: Chronic obstructive pulmonary disease
FEV-1: Forced expiratory volume, 1 second
RCT: Randomized controlled trial
