# Supplementary material for: Is the relationship among outcome variables shown in randomized trials?
Source: Trials. 2015 Feb 22;16:57. doi: 10.1186/s13063-015-0584-6 (PMC4374175; doi:10.1186/s13063-015-0584-6)
Supplement: Additional file 1: Table S1. — Impact Factor and CONSORT status of study journals. [file 13063_2015_584_MOESM1_ESM.doc]

Additional file 1: Table S1: Impact Factor and CONSORT status of study journals

| **Journal** | **2011 Impact Factor** | **CONSORT endorsement** |
| --- | --- | --- |
| **General Medical** |  |  |
| NEJM | 53.3 | Yes |
| Lancet | 38.3 | Yes |
| JAMA | 30.0 | Yes |
| Ann Internal Med | 16.7 | Yes |
| PLoS Medicine | 16.3 | Yes |
| BMJ | 14.1 | Yes |
|  |  |  |
| **Specialty Journal** |  |  |
| Am J Psychiatry | 12.5 | Yes |
| Ann Neurology | 11.1 | Yes |
| Ann Surgery | 7.5 | Yes |
| Radiology | 5.7 | Yes |
| Pediatrics | 5.4 | Yes |
| Obstet Gynecol | 4.7 | Yes |
| Ann Emerg Med | 4.1 | Yes |
|  |  |  |
| **Subspecialty Journal** |  |  |
| Circulation | 14.7 | Yes |
| Gastroenterology | 11.7 | Yes |
| J All Clin Immunol | 11.0 | Yes |
| Ophthalmology | 5.5 | Yes |
| J Am Acad Dermatol | 4.0 | Yes |
| Plast Reconstruct Surg | 3.4 | No |
| J Bone Joint Surg Am | 3.3 | Yes |
